# Canadian-led capacity-building in biostatistics and methodology in cardiovascular and diabetes trials: the CANNeCTIN Biostatistics and Methodological Innovation Working Group

**DOI:** 10.1186/1745-6215-12-48

**Published:** 2011-02-18

**Authors:** Lehana Thabane, George Wells, Richard Cook, Robert Platt, Janice Pogue, Eleanor Pullenayegum, David Matthews, Tara McCready, Philip J Devereaux, John A Cairns, Salim Yusuf

**Affiliations:** 1 Department of Clinical Epidemiology and Biostatistics, McMaster University, 1200 Main Street West, Hamilton, Ontario, L8N 3Z5, Canada; 2 Biostatistics Unit, St Joseph's Healthcare Hamilton, 50 Charlton Avenue East, Hamilton, Ontario, L8N 4A6, Canada; 3 The Centre for Evaluation of Medicines, St Joseph's Healthcare Hamilton, 105 Main St. East, Level P1, Hamilton, Ontario, L8N 1G6, Canada; 4 Population Health Research Institute, Hamilton Health Sciences, 237 Barton Street East, Hamilton, Ontario, L8L 2X2, Canada; 5 University of Ottawa Heart Institute, 40 Ruskin Street, Ottawa, Ontario, K1Y 4W7, Canada; 6 Department of Statistics and Actuarial Science, University of Waterloo, 200 University Avenue West, Waterloo, Ontario, N2L 3G1, Canada; 7 Department of Epidemiology and Biostatistics, McGill University, 1020 Pine Avenue West, Montreal, Quebec, H3A 1A2, Canada; 8 Department of Medicine, University of British Columbia, 2329 West Mall, Vancouver, British Columbia, V6T 1Z4, Canada; 9 Department of Medicine, McMaster University, 1280 Main Street West, Hamilton, Ontario, L8S 4L8, Canada

## Abstract

The Biostatistics and Methodological Innovation Working (BMIW) Group is one of several working groups within the CANadian Network and Centre for Trials INternationally (CANNeCTIN). This programme received funding from the Canadian Institutes of Health Research and the Canada Foundation for Innovation beginning in 2008, to enhance the infrastructure and build capacity for large Canadian-led clinical trials in cardiovascular diseases (CVD) and diabetes mellitus (DM). The overall aims of the BMIW Group's programme within CANNeCTIN, are to advance biostatistical and methodological research, and to build biostatistical capacity in CVD and DM. Our program of research and training includes: monthly videoconferences on topical biostatistical and methodological issues in CVD/DM clinical studies; providing presentations on methods issues at the annual CANNeCTIN meetings; collaborating with clinician investigators on their studies; training young statisticians in biostatistics and methods in CVD/DM trials and organizing annual symposiums on topical methodological issues. We are focused on the development of new biostatistical methods and the recruitment and training of highly qualified personnel - who will become leaders in the design and analysis of CVD/DM trials. The ultimate goal is to enhance global health by contributing to efforts to reduce the burden of CVD and DM.

## Background

Jointly funded beginning in 2008 by the Canadian Institutes of Health Research (CIHR) and the Canada Foundation for Innovation (CFI), the CANadian Network and Centre for Trials INternationally (CANNeCTIN) was among five successful applications under the CIHR-CFI strategic initiative to support clinical research programs, teams and network infra-structure that focused on high-impact, clinically relevant problems [1]. CIHR is the Canadian equivalent of the US National Institutes of Health. It was established by the Canadian federal government in 2000, as the successor to the Medical Research Council and currently supports more than 13,000 health researchers and trainees in universities, teaching hospitals and other health organizations and research centres across the country. CFI is an independent corporation created in 1997 by the Government of Canada to fund research infrastructure. The CFI's mandate is to strengthen the capacity of Canadian universities, colleges, research hospitals, and non-profit research institutions to carry out world-class research and technology development that benefits Canadians.

The focus for CANNeCTIN is to enhance the infrastructure and to build capacity for clinical trials in cardiovascular diseases (CVD) and diabetes mellitus (DM), especially those with a focus on neglected conditions, outcomes and interventions. There is an explicit goal to build capacity by developing young trialists who could emerge to be the leaders of large trials and expanding biostatistical expertise as it relates to clinical trials. Led by Salim Yusuf (Hamilton Health Sciences and McMaster University) and John Cairns (University of British Columbia), and coordinated by the Population Health Research Institute (PHRI) (Hamilton, Ontario), CANNECTIN has engaged more than 100 researchers from 19 universities including all of Canada's 17 medical schools. This network is linked to an international network of over 1500 affiliated hospitals and clinics from 80 countries [1] and is committed to developing links with investigators in developing countries (e.g. South Africa, other African countries, South American countries and India). PHRI was launched as a joint Hamilton Health Sciences and McMaster University research institute in 1999. It is located in the David Braley Cardiac, Vascular and Stroke Research Institute (DBCVSRI) at Hamilton General Hospital. The PHRI provides an education, training, and mentorship environment for learners working on cutting-edge projects with world class scientists, with a vision of world class research for new discoveries solving global health challenges. PHRI's research interests span all frontiers of the globe and include a broad spectrum of health-related issues. Originally a cardiovascular disease research institute, PHRI's programs have expanded to include a broad specrum of medical and societal conditions in varied populations defined by ethnicity or geographic region.

The organizational structure of CANNeCTIN includes Working Groups which are disease/discipline-based (e.g. interventional cardiology, perioperative medicine, cardiac surgery) and technological/programmatic in type (e.g., biostatistics and methodological innovation, pharmacogenomics, developing countries, education, knowledge translation (KT)) collaborative national and international networks and a coordination centre based within the PHRI [1]. A detailed description of the programme, organization, functions and goals is available at http://www.cannectin.ca.

At the planning stage of the CANNeCTIN program, it was recognized that biostatisticians play a critical role in designing health studies (by helping to formulate research questions and to determine appropriate research designs, data collection procedures and analytic strategies) and in conducting analyses of data from studies to answer scientific questions. The universal shortage of biostatisticians has been documented in many countries including the United States [2] and Australia [3]. In their "Strategy for patient-oriented research" released in February 2010, CIHR acknowledged that in Canada "...the clinical research workforce has not grown since CIHR's creation in 2000, with obvious shortages of *biostatisticians*, health economists, clinical epidemiologists, social scientists, ..." and further noted that "...most academic health science centres conducting clinical research report a critical shortage of *biostatisticians *and *methodologists*" (http://www.cihr-irsc.gc.ca) [emphasis is ours]. Canada needs to train and support the careers of biostatisticians and research methodologists to enhance its capacity to conduct patient-oriented research with the potential to improve health and enhance the sustainability of the Canadian healthcare system. A fundamental goal of CANNeCTIN is to improve this situation.

The objectives of this paper are: 1) to describe the innovative research and training activities of the Biostatistics and Methodological Innovation Working (BMIW) Group in building capacity in biostatistics and clinical trial methods; 2) to disseminate the activities of the BMIW Group to other interested researchers; and 3) to encourage interested biostatisticians and methodologically oriented clinical researchers to join the BMIW Group.

## The Biostatistics and Methodological Innovation Working Group

As one of the CANNeCTIN Working Groups, the BMIW Group was created to bring clinical researchers and biostatisticians together from multiple universities across Canada to: a) identify and address complex biostatistical and methodological problems relevant to clinical trials and epidemiologic studies; b) advise clinical trialists regarding these issues; and c) build capacity through practical and interactive training of future biostatisticians and clinician-methodologists in CVD/DM trials.

Innovations in biostatistical methods have already played a major role in health research; however, clinical researchers face increasingly challenging problems requiring further innovation. Unresolved biostatistical challenges include: a) optimal ways to analyze pharmacogenomic data; b) developing new statistical methods in the design and analysis of CVD/DM trials; c) addressing complexities in non-inferiority and adaptive trial designs. Using simulations and existing datasets from large completed studies (see PHRI website for a complete listing of completed and on-going multi-centre studies: http://www.phri.ca/body.cfm?id=22), the BMIW Group is able to empirically test their hypotheses on many of the above biostatistical and methodological issues.

**Figure 1:**
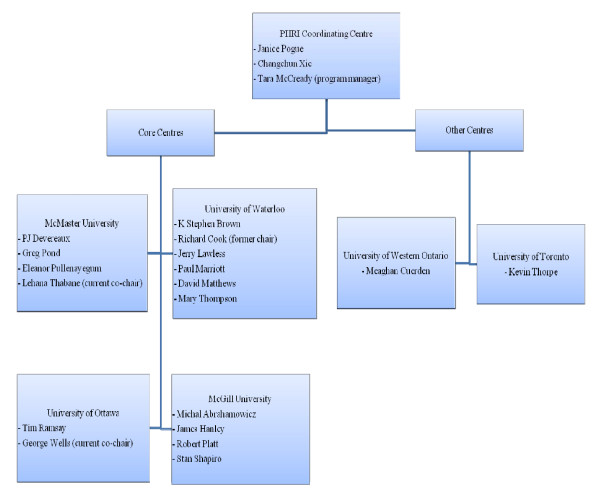
**Key Members of the CANNeCTIN Biostatistics and Methodological Innovation Group by Centre**.

The original core centres in the Group were the University of Waterloo, University of Ottawa, McGill University and McMaster University with PHRI as the coordinating centre. Other centres that have joined the Group include the University of Toronto and University of Western Ontario. Figure 1 shows the key members from each centre.

## Innovations in Biostatistical Research and Training

Our innovative program of research and training activities includes: monthly videoconferences on topical biostatistical and methodological issues in CVD/MD clinical studies; providing presentations on methodological issues at the annual CANNeCTIN meetings; recruiting and training young statisticians in biostatistics and methods in CVD/DM trials and organizing annual symposiums on topical methodological issues. These activities were deliberately chosen to share knowledge, provoke interactive discussion, stimulate research ideas, encourage research collaboration and cultivate academic cross-fertilization among biostatisticians, clinicians and trainees.

## Monthly video-conferences

The BMIW Group holds regular videoconference/webcast seminars on advanced issues in clinical trials methodology. The seminars take place every second Friday of the month between September to June. Participants can join the live videoconference sites at the Hamilton General Hospital, McMaster University, University of Waterloo, McGill University, and the University of Ottawa Heart Institute or view a live webcast of the presentation through the Ontario Telemedicine Network (OTN) website (http://webcast.otn.ca). The webcasts are archived on the OTN website for two years and then moved to the CANNeCTIN website. Individuals interested in taking part in the videoconferences can email cannectin@phri.ca for information about adding their centres to the live videoconference, join the videoconference in person at http://www.cannectin.ca/default.cfm?id=66 or watch a live webcast on the OTN website. The seminars have been running for three years and have covered a wide spectrum of issues in clinical studies (see Table 1). The pdf versions and some of the video webcasts of presentations are available online at http://www.cannectin.ca/default.cfm?id=25 and can form a useful learning resource for individuals both within t and outside of the network.

**Table 1:** Coverage of topics and issues at monthly videoconferences for 2008-2010

Topic	Issues covered*
Issues in RCTs	• Biases resulting from patient withdrawal in RCTs and how to address them;
	• Design and analysis issues in knowledge translation trials in primary care;
	• Testing for blinding at the end of an RCT [5];
	• Prognostic imbalance in RCTs;
	• Dynamic allocation methods: why the controversy?
	• Statistical issues in the use of composite outcomes clinical trials.

Issues in cluster RCTs	• Clustered measurement in cluster randomized trials;
	• Imputation strategies for missing binary outcomes in cluster randomized trials.

Non-inferiority designs	• What's wrong with non-inferiority designs?

Pragmatic Trials	• A pragmatic-explanatory continuum summary (PRECIS) [6];

Meta-analysis issues	• Empirical priors for between-study heterogeneity in meta-analysis;
	• Precision in meta-analysis;
	• Indirect comparisons for evaluating healthcare interventions [7].

Other issues	• Bias in logistic regression due to omitted covariates;
	• Statistical genetics and coronary artery disease;
	• Longitudinal modeling when the response and time-dependent covariates are measured at distinct time-points.

* Pdf Slides and audio versions of the presentations are available at http://www.cannectin.ca/default.cfm?id=25

## Presentations at CANNeCTIN annual meetings

To facilitate cross-fertilization between statisticians and clinicians, the BIMW Group regularly engages in interactive discussions with clinicians on methodological issues that arise in CANNeCTIN studies. Clinical investigators are invited to present to their studies to the group at our monthly biostatistics videoconferences and receive input from the group. The group members also give presentations at the annual CANNeCTIN meeting. At the CANNeCTIN Junior investigator meeting held in Winter 2010, the members of the group delivered the following presentations:

• How CANNeCTIN investigators can collaborate with statisticians at PHRI;

• What you need to know about pilot studies: the what, how and why [4];

• Some issues in the design and analysis of RCTs - covering issues in cluster randomization trials and time-to-event outcomes; and

• Common errors in CIHR RCT grants.

These presentations are available at http://www.cannectin.ca/default.cfm?id=116. We plan to continue to foster these interactions in future meetings and to expand the coverage to include more topics. The BMIW Group also participated in the Cutting Edge Symposiums on Pharmacogenomics that took place in May 2009 and Perioperative Medicine held in April 2010, in Hamilton (Ontario, Canada) (http://www.cannectin.ca/body.cfm?id=68). In these symposiums, we collaborate closely with our clinician investigators to discuss the biostatistical and methodological issues arising from different studies. For example, one of the trials - PADIT (Prevention of Arrhythmia Device Trial) [ClinicalTrials.gov identifier #: NCT01002911] involved both the use of both a cross-over design and cluster-randomization. The trial uses "site" as a unit of randomization. The primary objective is to compare conventional pre-operative antibiotics versus conventional intra-operative plus post-operative antibiotics in preventing hospitalization attributed to device infection in patients undergoing cardiac surgery. This raised interesting discussions among biostatisticians and clinicians about the design and analysis of the trial.

The group also takes part in the annual Trout-CANNeCTIN workshop - an intensive, interactive training program led by Dr David Sackett, which guides young researchers in the design, implementation, and analysis of high-quality clinical studies, as well as in effective grant writing and academic career development. Our role includes providing biostatistical feedback and support to the trainees.

## Capacity-building through training and mentorship of young biostatisticians

One of our goals is to build capacity in biostatistics within Canada through advanced and practical training of graduate and postgraduate students. This occurs in an interdisciplinary environment, with clinicians and biostatisticians working together on applied problems. As noted earlier, the worldwide need for experienced biostatisticians in many countries is high in all areas of health research [2,3] and Canada is no exception. Our program is to recruit and train biostatisticians within Canada with the necessary practical experience and skills to work with clinical investigators. The specific objectives of this training and mentorship programme are to promote enthusiasm and commitment to excellence in statistical collaboration in CVD and DM clinical research; to enhance communication of statistical issues to clinician collaborators; to train young statisticians to acquire biostatistical self-sufficiency and develop skills in applied statistics in CVD and DM trials; and to enhance a culture of collaboration among statisticians and clinician researchers. We introduce our trainees to the rewards and challenges of health care research, through practical training obtained by working on real problems that are encountered in ongoing trials. We provide partial funding for all CANNeCTIN biostatistics trainees, who have a primary supervisor at their "home" institution. All supervisors have to be active members of the BMIW Group. The student submits a proposal and receives input from all the members of the group (collective mentoring). By providing hands-on experience in large clinical studies, our training approach complements training programs already existing in many universities and builds biostatistical capacity specific to CVD and DM trials in Canada.

Table 2 provides a summary of our trainees to date (11 trainees), their "home" institution, project title and primary supervisor(s).

**Table 2:** Current List of CANNeCTIN Biostatistics Trainees

Institution	Trainee	Project	Supervisor
McMaster University	Kristian Thorlund	Methodological improvements in meta-analysis	Drs. Lehana Thabane and PJ Devereaux

	Jinhui Ma	Issues in the Statistical Analysis and Imputation Strategies for Binary outcome from Cluster Randomized Trials in Management of Cardiovascular Risk Factors	Dr. Lehana Thabane

	Rachel Chu	Intraclass correlation in multicentre RCTs and prognostic heterogeneity/imbalance	Dr. Lehana Thabane

	Hoi Suen	Generalised Additive Models for the analysis of health utility data	Dr Eleanor Pullenayegum

University of Waterloo	Haocheng Li	Design of Clinical Trial and Statistical Analysis with Missing Data	Dr. Grace Yi

	Longyang Wu	Design of Clinical Trials with Recurrent Events and Competing Risks	Dr. Richard Cook

	Audrey Boruvka	Event history analysis with incomplete data	Dr. Richard Cook

	Min Chen	Empirical Likelihood Methods for Pretest-Posttest Studies	Drs Mary Thompson and Changbao Wu

University of Ottawa	Robert William Davies	Statistical Issues Which Pertain to the use of GWAS for the Identification, Characterization and Quantification of Genetic Effects in Cardiovascular Disease	Dr. George Wells

McGill University	Maria Esther Perez Trejo	The problem of extra variation induced by double clustering	Dr. Robert Platt

	Michael Regier	Develop causal methods appropriate for repeatedly measured data	Dr. Robert Platt

	Menglan Pan	Marginal Structural Models, Odds Ratios, and Collapsibility	Drs. Robert Platt and Jay Kaufman

## Other Activities: Special Invited Sessions at Scientific Meetings and the Annual Biostatistics and Methods Symposium

The BMIW Group has organized some special sessions sponsored by CANNeCTIN at various biostatistical meetings including the Canadian Society for Epidemiology and Biostatistics annual meeting held in Ottawa (Ontario, Canada) in, 2009; and the Statistical Society of Canada annual meeting held in Vancouver (British Columbia, Canada) in 2009. We are currently organizing another invited sessions at the annual meetings of the Statistical Society of Canada (SSC) in Wolfville (Nova Scotia, Canada) in June 2011 and International Society for Clinical Biostatistics (ISCB) in Ottawa (Ontario, Canada) in August 2011. These activities have provided good forums for the group to share their research with national and international statistical communities

The BMIW group plans to organize annual symposiums on challenging and relevant topics in biostatistics in RCTs or observational studies that will involve other experts from Canada and abroad, and be suitable for publication in peer-reviewed journals. Each symposium will focus on answering four key questions: 1) what is the state-of-the-art? ii) what are the outstanding or controversial issues that remain unresolved or need to be addressed? iii) what guidance can we provide to researchers on these issues based on the current knowledge?; and iv) what research needs to be done to fully address the issues?

Topical biostatistical and methodological issues that have been identified so far include composite outcomes, stopping rules, surrogate outcomes, non-inferiority designs, non-compliance, adjustment for baseline imbalance in RCTs, adaptive designs, and large vs. small trials. The first CANNeCTIN Cutting Edge Symposium on Advanced Biostatistics and Methodological Issues in Clinical Trials is planned for Hamilton, Canada for April 28-29, 2011, will focus on outcomes in CVD trials - covering both composite and surrogate outcomes, and adaptive designs. More details on the symposium can be found at http://www.cannectin.ca/default.cfm?id=128.

## Lessons Learned

Effective use of information technology (IT) to link different centres through videoconferences has been key to the success of our monthly seminars. Occasionally, we encounter some technical problems in connecting sites, but these problems are always resolved without much difficulty and with some patience. To date, no seminars have been cancelled because of IT challenges.

Mentoring the next generation of biostatisticians in cardiovascular trials has been a worthwhile journey so far. Collectively, we have 11 trainees from four Canadian universities, supervised or mentored by seven BMIW Group biostatisticians. We hope that this effort will continue to expand the training opportunities to more national and international centres.

Interacting and collaborating with clinicians in addressing CVD/DM problems has been a rewarding experience for many of us. The CANNeCTIN programme provides an excellent opportunity to promote academic dialogue among biostatisticians and clinicians (including our trainees). By sharing knowledge, provoking interactive discussion, stimulating research ideas, encouraging collaboration and fostering academic cross-fertilization, we can make our biostatistical research more relevant and applicable to important problems in CVD and DM research.

## Some Concluding Remarks

There is a severe shortage of biostatistical expertise in CVD/DM research. Our efforts to reduce the global burden of these diseases depend on the conduct of large well-designed trials to advance our understanding of the key predictors of the disease and the best strategies for prevention, treatment and management. The BMIW Group is making substantial contributions towards the realization of CANNeCTIN's vision to advance global health through building the capacity of the Canadian research community to lead major CVD and DM clinical trials. The long-term contributions of the BMIW Group will include the development of new biostatistical methods and recruitment and training of highly qualified personnel. These personnel will become leaders in collaborative health research through contributions to the design and analysis of CVD/DM trials, and also serve as committee members and reviewers for granting agencies such as CIHR that support clinical research in Canada. We believe that with suitable investments, this is a model that can be useful for building human capacity in other areas of clinical research experiencing similar shortage of biostatistical expertise.

## Competing interests

The authors declare that they have no competing interests.

## Authors' contributions

LT drafted the manuscript. All authors reviewed and edited draft versions of the manuscript and approved the final version.
